# Porous deproteinized bovine bone scaffold with three-dimensional localized drug delivery system using chitosan microspheres

**DOI:** 10.1186/s12938-015-0028-2

**Published:** 2015-04-15

**Authors:** Qing Li, Gang Zhou, Xin Yu, Tong Wang, Yuan Xi, Zhihui Tang

**Affiliations:** Center of Digital Dentistry, Peking University School and Hospital of Stomatology, 22 Zhongguancun Nandajie, Haidian District, Beijing, 100081 China; National Engineering Laboratory for Digital and Material Technology of Stomatology, 22 Zhongguancun Nandajie, Haidian District, Beijing, 100081 China; Key Laboratory for Biomechanics and Mechanobiology of Ministry of Education, School of Biological Science and Medical Engineering, Beihang University, Beijing, 100191 China

**Keywords:** Deproteinized bovine bone (DBB), Scaffold, Microsphere, Drug delivery system

## Abstract

**Background:**

Bone substation grafts, such as hydroxyapatite (HA) and tricalciumphosphate (TCP), have been extensively used in clinical applications, but evidence suggests that they offer poor osteoinductive properties compared to allografts and autografts. In order to increase bone growth with such grafts, Bone Morphogenetic Protein 2 (BMP-2) was incorporated into a three dimensional reservoir. The purpose of the present study was to develop a novel drug delivery system which is capable of controlled release of BMP-2.

**Methods:**

DBB were prepared from bovine cancellous bone harvested from fetal bovine femur or tibia and then sinting at 1000°C. BMP-2-loaded chitosan (CS) microspheres were fabricated by cross-linking. Then the treated DBB powders were blended with chitosan microspheres solution. Finally, the composites were lyophilized with a freeze dryer to obtain the DBB/CMs scaffolds. X-ray diffractor (XRD), scanning electron microscopy (SEM) and Fourier transform infrared (FT-IR) were used to characterize the sample. The quantification of the delivery profile of BMP-2 was determined using an enzyme-linked immunosorbent assay (ELISA) kit. The in vitro assays were to characterize the biocompatibility of this composite.

**Results:**

In this study, BMP-2/Chitosan (CS) microspheres were successively loaded onto a deproteinized bovine bone (DBB) scaffold. The release profile of BMP-2 indicated an initial burst release followed by a more even sustained release. An in vitro bioactivity assay revealed that the encapsulated growth factor was biologically active.

**Conclusions:**

The cell culture assay suggest that the excellent biocompatibility of the DBB- BMP-2/CS. Therefore, this novel microsphere scaffold system can be effectively used in current tissue engineering applications.

## Background

An increasing number of patients suffer from bone defects that require transplantation [1]. However, bone grafting, the gold standard in bone defect management, bears considerable risks; donor site pain and morbidity, infection, increased blood loss, and longer operating times [2-4]. The most common alternative is to use human cadaveric bone (allograft), which has additional disadvantages, including rejection, limited supply, and potential disease transmission. Interest has thus focused on animal bone (xenograft) that exhibits physiological and chemical properties similar to autologous bone. But inherent complications with immunogenicity and disease transmission also hinder the effectiveness of this method [5].

For an ideal bone tissue engineering scaffold, one of the most important criteria is that the scaffold consists of a highly interconnected porous network with pore sizes large enough for cell migration, fluid exchange, and eventually tissue ingrowth and vascularization. Although synthetic calcium phosphate ceramics (such as β-tricalciumphosphate (β-TCP) [6], and hydroxyapatite (HA) [7]) with their excellent biocompatibility are designed to mimic the native extracellular matrix as closely as possible [8], the porous network of the artificial bone scaffold still cannot compare to natural bone in terms of structure and function.

There is a growing need to provide alternatives to traditional bone grafting. Deproteinized bovine bone (DBB) [9] manufactured from natural materials, such as coral or natural bone, has the advantage of inheriting the properties of the raw source materials, including the pore structure. Because all organic material is removed by stepwise annealing (up to 300°C), there is no need for concern with immunogenicity or disease transmission. Therefore, this kind of inorganic porous bone has gained wide acceptance for various dental and medical applications; e.g. fillers for periodontal defects, alveolar ridge augmentation, maxillofacial reconstruction, dental implants and spine fusion. DBB has been widely used in dentistry as an alternative to autologous bone grafts. Although DBB provides an osteoconductive scaffold, it is not capable of enhancing bone regeneration because it is not osteoinductive.

In addition, during the bone healing process, the constant remodeling activities are coordinated and controlled by various growth factors, such as TGF-β, PDGF, FGF-2, and BMPs [10]. The osteoinductive capacity of bone morphogenetic proteins (BMPs), especially BMP-2, have been proven to promote osteogenic differentiation [11]. BMP-2 is now approved by the U.S. Food and Drug Administration (FDA) for restricted clinical use in open tibial fractures and anterior spinal fusion. In order to render DBB osteoinductive, bone morphogenetic protein 2 (BMP-2) has previously been incorporated into a three dimensional reservoir (a biomimetic calcium phosphate coating) on DBB, which effectively promoted the osteogenic response by the slow delivery of BMP-2. Despite the proven efficacy of BMP, its clinical application is complicated by its short biological half-life, systemic side effects and rapid clearance [12]. Delivery systems that minimize BMP diffusion away from its therapeutic target are desirable not only for enhancing bone formation, but also for limiting unwanted pathologies.

In order to retain the BMPs at the site for a prolonged period of time [13], delivery via microspheres synthesized by chitosan may be used. Chitosan has been widely used for the controlled delivery of peptides or proteins in the form of microspheres, due to its excellent biological properties such as biodegradability, biocompatibility, non-toxicity, bacteriostaticity and strong adhesion [14]. The chitosan used for the synthesis of these microspheres can be both degradable [15] as well as non-degradable [16]. Incorporation of the molecules in these microspheres allows controlled diffusion and exposes the protein molecule for a longer duration.

In this study, deproteinized bovine bone (DBB) and chitosan were chosen as carriers for BMP-2 delivery to overcome the disadvantage of rapid clearance typically associated with BMP-2. Firstly, chitosan microspheres (CMs) loaded with BMP-2 were prepared by an emulsion-ionic cross-linking method. Secondly, CMs with BMP-2 was embedded into DBB by a dip-coating process using the capillarity of the material. The purpose of this study was to increase the efficiency of this microsphere-scaffold as a carrier for growth factors and to estimate the osteogenic effect of this microsphere-scaffold as a potential candidate for bone tissue engineering.

## Materials and methods

### Preparation of the DBB scaffold

Samples were prepared from bovine cancellous bone harvested from fetal bovine femur or tibia. Using a diamond saw, the cancellous bones were sectioned into 3 mm × 3 mm × 40 mm blocks. The fatty bone marrow was removed by immersion in 30% H_2_O_2_ for 2 h. The bone was washed with water and immersed in 1% H_3_PO_4_ for heating in a water bath at 125°C for 2 h. The bone was washed with 95% alcohol to remove any residual moisture that could cause small cracks during heat treatment. Following air drying for at least 12 h, the bone underwent heat treatment in a furnace (Siliconit muffle furnace, Kwangsung Science Co., Korea) with a heating rate of 10°C/min up to 1000°C. This temperature was maintained steady for 2 h under 1 atm of pressure. To obtain constant size particles of 0.25-1 mm and 1-2 mm, a sieve with corresponding mesh size was used. After ultrasonic cleaning and drying, the deproteinized bovine bones were obtained.

### Preparation of BMP-2 loaded chitosan microspheres

BMP-2-loaded chitosan (CS) microspheres were fabricated by cross-linking. Briefly, CS polymer with a viscosity of 150–900 cP and 75–85% deacetylation was purchased from Sigma-Aldrich (Shanghai, China), and then refined twice by dissolving in aqueous acetic acid solution and precipitating from dilute ammonia. The BMP-2 was also obtained from Sigma-Aldrich. A CS solution (10 g/l) was prepared by dissolving CS in 2.3% (v/v) aqueous acetic acid solution. 500 mg of this CS solution and the different contents of BMP-2 (0.75 μg/ml, 1 μg/ml, 1.25 μg/ml) were mixed and then the cross-liker was added (vanillin 1% v/v ethanol aqueous solution). The emulsified solution was immediately poured into the CS-BMP-2 mixture and stirred with a magnetic stirrer for 4 h so as to allow the solvent to evaporate. The hardened microspheres were then collected by centrifugation at 3000 rpm for 3 min, washed three times with distilled water, and lyophilized using a freeze dryer.

### Preparation of DBB/CMs scaffold

Preparation of BMP-2-loaded CS microspheres on DBB scaffold surfaces was performed in the following process. In the first-step, DBB powders were radiofrequency (RF) plasma glow-discharged (PDC-32 G, Harrick Plasma, USA) in an oxygen-filled chamber at a pressure of 200 mTorr Pa. The plasma power density and the treatment time were fixed at 30 W and 120 s, respectively. Then the treated DBB powders were blended with different contents of chitosan microspheres solution and poured into a polystyrene mold, and frozen at −20°C overnight. Finally, the composites were lyophilized with a freeze dryer to obtain the DBB/CMs scaffolds.

### Characterization of materials

XRD was used to determine the structure of the synthesized DBB. XRD measurements were performed with an X-ray diffractor [D/max-II; X-ray Diffractometer (XRD), RIGAKU, Japan] (Cu-Ka). The samples were measured in the 2θ range from 10° to 70° (scan speed of 0.02° per second).

The morphology of the obtained chitosan microspheres and DBB scaffolds was examined by a scanning electron microscopy (SEM, S-2400; Hitachi, Japan).

Chemical analysis of the DBB, CS and DBB/CMs materials was carried out by a Fourier transform infrared (FT-IR) spectrophotometer (FTIR 650) in the range from 4000 cm^−1^ to 400 cm^−1^ at a resolution of 3 cm^−1^, averaging 100 scans.

### In vitro release profile

The quantification of the delivery profile of BMP-2 (0.75 μg/ml, 1 μg/ml, 1.25 μg/ml) from this system was determined using an enzyme-linked immunosorbent assay (ELISA) kit (PeproTech EC, London, UK) [17].

### In vitro study

Mouse calvaria-derived, pre-osteoblastic cells MC3T3-E1 were cultured as monolayers in Alpha Minimum Essential Medium (α-MEM, GIBCO), supplemented with 10% (v/v) fetal bovine serum (FBS; Sigma), 1% (v/v) L-glutamine (Invitrogen Corp., Carlsbad, CA, USA), and a 1% (v/v) antibiotic and antimycotic formulation (containing penicillin G sodium, streptomycin sulfate (Invitrogen Corp., Carlsbad, CA, USA). The medium was changed on alternate days, and the cultures were maintained at 37°C within a humidified atmosphere containing 5% CO_2_.

When the confluence reached 90%, the cells were passaged (P1). After passaging three times (P3), the cells were exposed to proliferation assays using CCK8 kits at 3d, 5d, 7d, 14d to study the impact of BMP-2 on the multiplication of MC3T3 cells. The cells were assigned into BMP-2 groups (0.75 μg/ml, 1 μg/ml, 1.25 μg/ml) and a control group.

Each cell suspension (5 ml) was plated at a density of 1 × 10^6^ cells ml^−1^ in plates containing sterile samples of microsphere-scaffolds for the cell attachment assays. SEM micrographs were taken at 1 day following the initial cell co-culturing with the scaffolds. Briefly, the co-cultured constructs were harvested, washed with PBS and then fixed with 4% glutaraldehyde. Following 3 rinses with water, these samples were dehydrated through a series of graded alcohol solutions and then air-dried overnight. The samples were observed by SEM at an accelerating voltage of 30 kV (QUANTA 250 FEG, USA).

### Differentiation of cells on microsphere-scaffold

MC3T3-E1 cells were seeded with new medium. When the density reached 10% after 3 passages, about 1 × 10^6^ cells were seeded on T25 flasks. Then these colonies were switched to an osteo-inducing medium (a-MEM, 10-8 M dexamethasone, 50 μg/ml ascorbic acid, 10 mM ß-glycerophosphoric acid). The cells were assigned into a BMP-2 group and control group respectively. Samples from each group were collected at 3d and 7d for western blotting and real-time PCR analysis.

### Western blotting

MC-3 T3 cells were lysed in an RIPA buffer supplemented with protease inhibitors (#11-10601, SinoGene Scientific, Beijing China) and phosphatase inhibitors (#11-10701, SinoGene Scientific, Beijing China). Protein concentration was determined using the Bradford method. 30 μg protein lysate was mixed with 2× loading buffer at 1:1 and heated for 10 min at 95°C before being loaded for gel electrophoresis.

The proteins and protein marker (SM1881, Thermo Fermentas) were separated by SDS-PAGE (4% stacking gel, 12% separating gel) at 120 V for 2 h. Proteins were transferred to PVDF membranes using a Bio-rad mini transfer system. The membranes were blocked in rapid blocking Buffer (#66-11402, SinoGene) for 5 min and then probed with primary antibodies against ALP, BMP-2, OST and RUNX2 at a dilution of 1:1000 for 2 h. After washing 3 times with PBS, at 10 min per wash, HRP-conjugated anti rabbit and anti-mouse antibodies (10104, 10105, Miroanalysis Inc.) were applied to the membranes at a dilution of 1:2000 for 1 h. Chemiluminescent signals were developed using a ECL Kit (ECL, 29050, Engreen) and exposed to X-ray films in a dark room. Then the membranes were stripped by a stripping buffer (#66-11501, SinoGene Scientific) and reprobed with ß-actin (M20010, Ab-mart). The level of actin was measured as an internal control.

### Real-time PCR analysis

Total RNA was isolated from samples using an SG TriEx HiPure RNA Extraction Kit (#55-11120, SinoGene Scientific, China). RNA concentration was measured at 260 nm and its purity was assessed with Biophotometer (Eppendorf, Germany) at a ratio of 260:280 nm. RNA samples were treated with DNaseI (EN0523, Fermentas) to remove contaminating genomic DNA and analyzed on 1.5% agarose gels. Reverse transcription was performed with a Thermo First cDNA Synthesis Kit (#33-20102, SinoGene Scientific China) with random hexamer primers. The average amount of RNA used in the reverse transcription reaction was 1 μg and its purity was around 2.0 (260:280 nm ratio).

qPCR per tube contained 10 μl of 2 × SG Green qPCR Mix (with ROX), (#22-10102, SinoGene Scientific, China), 250 nM of each primer (List of primers as Table 1), PCR grade water and 1 μl of cDNA template (diluted in a 1:2 or 1:10 ratio). All samples were assayed in duplicates with each run. The PCR cycling program consisted of 10 min at 95°C, followed by 40 cycles of 15 s at 95°C and 60 s at 58°C. An additional step was used (95°C for 15 s, 58°C or 55°C for 30 s and 95°C for 15 s) for dissociation curve analysis. Data were analyzed by 2-deltadelta Ct method; a statistical comparison between samples was performed using unpaired Student’s *t*-test. A p value of <0.05 was considered statistically significant.

**Table 1:** **Chain reaction primers used for detection in this study**

**Primers sequence**	**Target gene**	**Product size (bp)**
CGACAGCAAGCCCAAGAG	ALP	110
GTGGAGACGCCCATACCA		
ACATCCGCTCCACAAACG	BMP-2	132
GGTGCCACGATCCAGTCA		
CTTCTCAGAGCCTCAGTCC	OCN	129
ACCGTAGATGCGTTTGTAG		
CTGGCGGTGCAACAAGAC	RUNX2	136
AACAGCGGAGGCATTTCG		
ACTCGCTGCGCTCGGTCGTT	ACTIN	125
CCTTTTGCTGGCCTTTTGCTCAC		

### Ethics statement

All experiments involving the use of animals were incompliance with Provisions and General Recommendation of Chinese Experimental Animals Administration Legislation and were approved by Beijing Municipal Science & Technology Commission (Permit Number: SCXK (Beijing) 2006-0008and SYXK (Beijing) 2006-0025).

### Statistical analysis

Data is given as means ± S.E.M. for statistical comparison, *t*-test or one-way ANOVA followed by Tukey’s test was used.*p < 0.05, **p < 0.01 and ***p < 0.001 were considered to be statistically significant.

## Results

### XRD measurement

**Figure 1:**
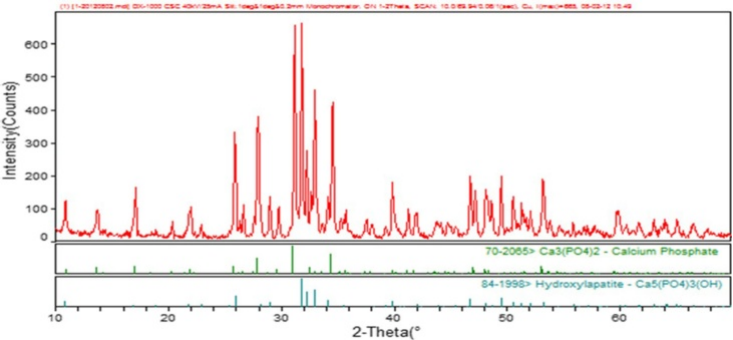
XRD pattern of DBB.

The XRD pattern of DBB is shown in Figure 1. The XRD patterns of the DBB samples (Figure 1) are very similar to that of pure HA and TCP, and are in accordance with ASTM data (Card 84–1998 and 70–2005). All of the diffraction peaks were sharp and well resolved, indicating the two obtained phases.

### SEM of CMs

**Figure 2:**
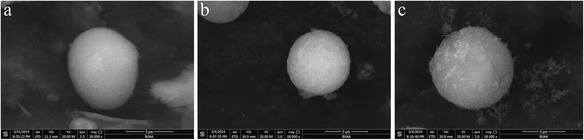
SEM of CMs (**a**: 1.25 μg/ml BMP-2; **b**: 1 μg/ml BMP-2; **c**: 0.75 μg/ml BMP-2).

The microspheres were spherical and had a regular surface (Figure 2). The particle size distribution was in the range of 9.634 μm, based on SEM observations. In this study, the vanillin concentration is crucial to the morphology of the microspheres. To prepare spherical microspheres with smooth surfaces, the vanillin concentration should be 1% so assist with crosslinking the CS molecules.

**Figure 3:**
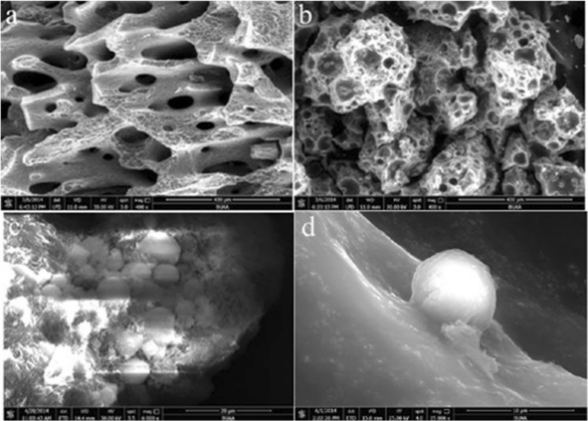
SEM of DBB (**a**: amplified × 400) and DBB/CM/BMP-2 composite scaffold (**b**: amplified × 600; **c**: amplified × 4000; **d**: amplified × 15000).

The surface morphology and interconnectivity of the fabricated scaffolds were analyzed. Figure 3a presents a surface image of DBB showing a densely porous structure with good interconnectivity between the pores. After loading the CMs, the DBB/CMs/BMP-2 scaffold presented smaller pores with reduced tunnel sizes for interconnectivity (shown in Figure 3b) than that of the pure DBB scaffold. It also demonstrated that DBB succeed loading the CMs. The pore size of the DBB scaffold was focused in the range from 300 to 400 mm. However, as depicted in Figure 3b, the pore size of the DBB/CMs/BMP-2 scaffold had a wider range from 80 to 300 mm. This greater pore size distribution could affect cell-proliferation. The stability of the microspheres on the surface of DBB scaffold was shown in Figure 3c,d, respectively. SEM revealed the microspheres bonded with DBB by plasma modified. These immobilized microspheres help the release profile from microspheres immobilized on DBB scaffold surfaces.

### FT-IR analysis

**Figure 4:**
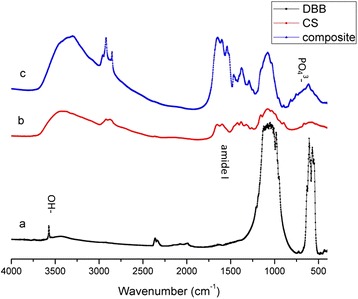
FT-IR of CMs (**a**: DBB; **b**: CS; **c**: DBB/CMs/BMP-2 composite).

IR spectra of synthesized DBB, pure CS, and DBB/CMs/BMP-2 composite powders are shown in Figure 4a,b and c. The major DBB bonds, which are associated with PO_4_^3−^ (1035, 963 and 604 cm^−1^) and OH^−^ (3571 cm^−1^), are apparent in Figure 4c. IR spectra show that there were some changes in the frequencies of PO_4_^3−^ and OH^−^, which shift to low wave numbers in the composite. In addition, the bonds of CO_3_^2−^ (1422 and 1484 cm^−1^) and HPO_4_^−^ (871 cm^−1^) also appear in the spectra (Figure 4a and d). These ions also show up in natural bone apatite, called non-stoichiometric apatite.

In Figure 4b, the characteristic peaks of pure CS are displayed at 1655 cm^−1^, which can be assigned to the absorption of amide I carbonyl stretching, while the peak of carbonyl stretching shifted to 1643 cm^−1^ in Figure 4c. This change signifies interaction between CS and DBB.

### Release testing

**Figure 5:**
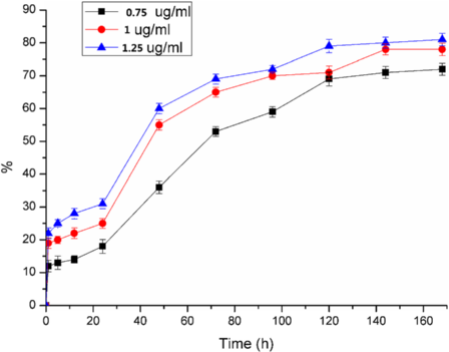
Release profile for BMP-2 from DBB/CMs/BMP-2 scaffolds in PBS within 7 days.

Figure 5 shows the in vitro release profiles of encapsulated BMP-2 from CS microspheres loaded on the DBB scaffold. An early burst of approximately 19% of the total BMP-2 can be seen after being loaded for 24 h, followed by a sustained release of the remaining BMP-2 over the next 7 days. The experimental data curve indicates that the BMP-2 release behavior might be attributed to both a reaction-controlled mechanism and diffusion-controlled mechanism. Thus, this synthesized DBB/CMs/BMP-2 system would allow for intercelluar release of the drug with high local concentrations at the site of interest.

### Attachment and proliferation of cells on DBB/CMs/BMP-2 scaffold

**Figure 6:**
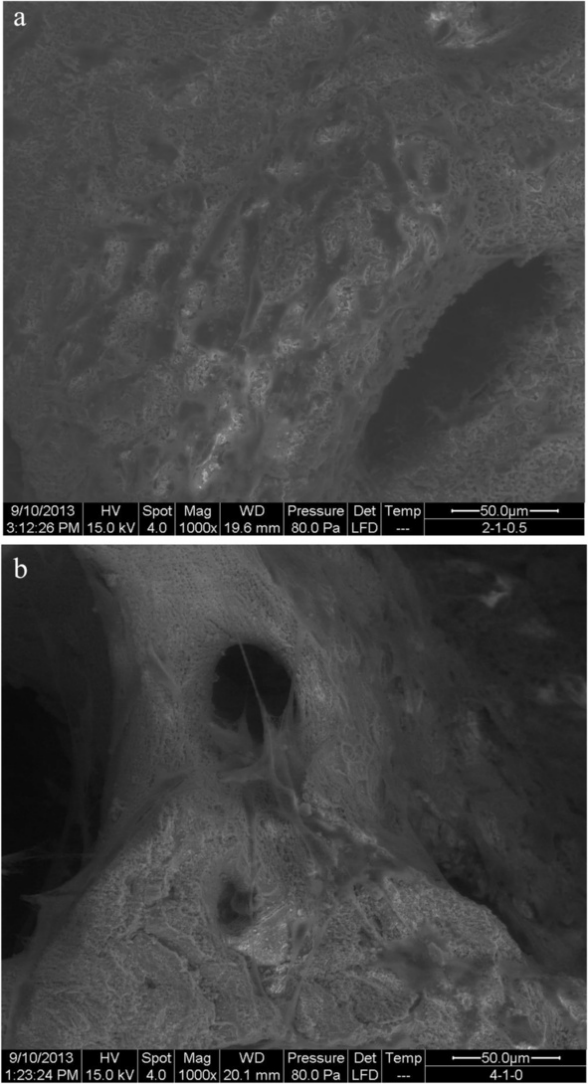
SEM of cell growth on the material surface (**a**: control without BMP-2; **b**: DBB/CMs/BMP-2 scaffold).

SEM images displaying the morphological features of pre-osteoblastic cells MC3T3-E1 cultured on the bone particles for 24 h are shown in Figure 6. The cells were observed to spread well and had intimate contact with the inner surface of the scaffold (Figure 6a). Upon examining the scaffolds at an increased depth, the cells could be seen to adhere tightly to the crystalline structure of the bone, with aggregated cells being clustered along the pore walls of the scaffolds (Figure 6b).

**Figure 7:**
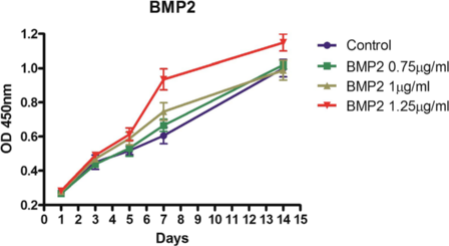
The proliferation of cells on CMs- scaffold with BMP-2.

A CCK-8 assay was used to evaluate the cytotoxicity of the CMs- scaffold, and the OD values can provide an indication of cell growth and proliferation on various materials. As seen in Figure 7, the OD values for the BMP-2 scaffold increased with culture duration, which indicates that this particular scaffold had no detrimental effect on the viability and proliferation of MC3T3-E1 cells, thus demonstrating good biocompatibility. In addition, this trend suggested that increasing BMP-2 concentration promote cell proliferation in these 7 days.

### Differentiation of cells on CMs- scaffold

**Figure 8:**
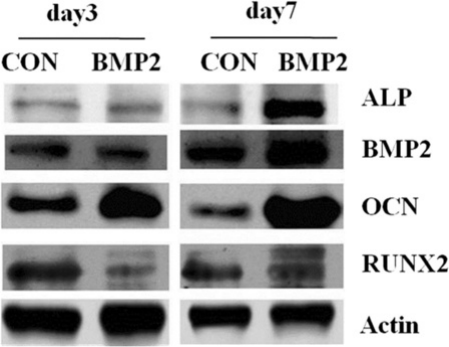
On the 3rd day, the expression levels of OCN and ALP were increased and RUNX2 was decreased in contrast with the control group, while the expression of BMP-2 had no significant difference between the two groups. On the 7th day, the expressions of ALP, OCN, BMP2 had increased and the expression of RUNX2 had decreased in both the control and BMP-2 group compared with their expression status on the third day.

**Figure 9:**
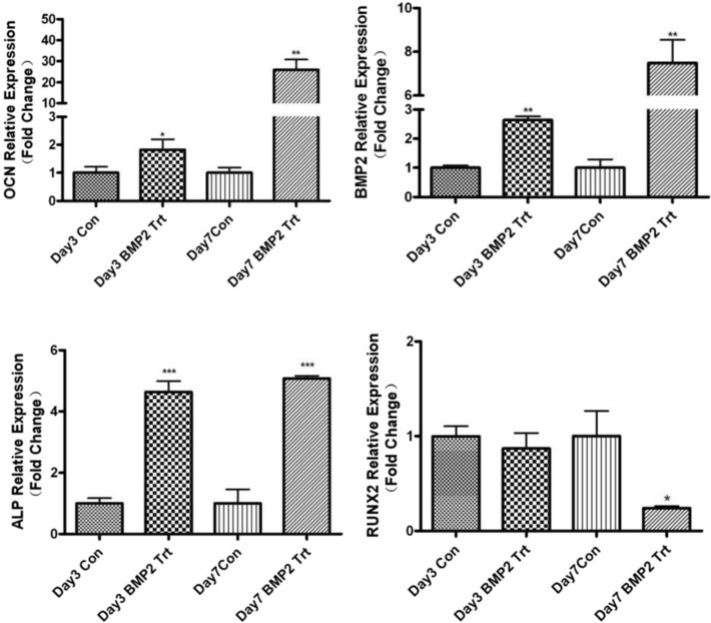
Compared the control group, on the 3rd day and the 7th of treatment, expression levels of genes OCN, BMP-2 and ALP increased (p < 0.05). RUNX2 genes were observed to decrease.

Histochemical analyses revealed strong osteogenic properties for the BMP-2 containing CM scaffold. In order to further confirm the differentiation of MC3T3-E1 cells, the expression of ALP, BMP-2, OCN and RUNX2 was analyzed at the 3rd day and the 7th day after culturing on induced medium by western blot (Figure 8). Inductions of osteoblast specific genes ALP, BMP-2, OCN, and RUNX2 were examined by RT-PCR (Figure 9). The results of qPCR were consistent with the WB analysis, which showed that the expressions of OCN, ALP and BMP-2 were increased in contrast with the CON group. The fold-change of gene expression in the BMP-2 treatment data is shown in Table 2.

**Table 2:** **Fold-change of different gene expressions after BMP-2 treatment**

	**Day3 CON**	**Day3 Trt**	**Day 7 CON**	**Day 7 Trt**
ALP	1 ± 0.1716	4.637 ± 0.3565***	1.003 ± 0.4517	5.077 ± 0.08647***
BMP-2	1 ± 0.08	2.64 ± 0.13**	1 ± 0.2894	7.483 ± 1.058**
OCN	1.003 ± 0.2128	1.823 ± 0.3724*	1 ± 0.1856	25.94 ± 4.835**
RUNX2	0.9967 ± 0.1073	0.87 ± 0.1626	1 ± 0.2649	0.2404 ± 0.02242

***P < 0.001, **P < 0.01, *P < 0.05.

Osteoblast cells were noted to proliferate on the materials on day 7 of culture; and the osteoblasts in the DBB/CMs scaffold without BMP-2 showed a lower increase on the 7th day of culture compared with those cultured on the other days. In contrast, cells growing on the DBB/CMs/BMP-2 scaffold did not show an increased tendency for proliferation at the same time point either with BMP-2. According to the data of the proliferation assay, the osteoblast cells in the DBB/CMs/BMP-2 scaffold had higher proliferation rates within 7 days than the controls, which indicated that the BMP-2 promoted cell proliferation. The osteoblast cells in this novel drug delivery system reached the end of first period faster than the control, which implied that the applied BMP-2 was beneficial to the osteoblast cells’ differentiation as well. Hence, Figure 9 not only shows the tendency for osteoblast proliferation induced by the drug delivery scaffold with or without BMP-2, but also indirectly reflects osteoblast differentiation.

## Discussion

The main objective of this study was to investigate BMP-2/CMs scaffolds as potential dual-drug/protein delivery systems for slow and sustained delivery of different bioactive agents. The retrieved data confirmed that a gradual, sustained, and cell-mediated release of bioactive agents can be achieved in vitro using a BMP-2/CMs scaffold. Moreover, the DBB/CMs scaffold loaded with BMP-2 showed excellent osteoinductivity.

In designing a matrix for differentiation factor release, it is apparent that the extremes of release (bolus injections or prolonged low level release) are not beneficial to bone induction. A further complicating factor is that different anatomical sites might require different kinetics of release for optimal performance. For example, in more fluid environments or compromised (avascular) sites, BMP clearance might be faster than the bone-induction response of the host. In these cases a slow-release system may be required. It has been suggested that bone repair therapy is influenced not just by the amount of protein present but also by the duration of protein production. So an important issue when accessing growth factors for local bone repair is to identify an ideal drug delivery carrier which can ensure a sufficient protein concentration and effect at the application site for the duration of the healing process, and therefore provide an appropriate support for bone repair. In the current study, the BMP-2 release resulted in a gradual, sustained protein release pattern. Further investigations are needed to analyze the in vivo release of BMP-2.

The degradability of CaP-based materials is very important for the in vivo longevity and efficacy of its biological effects. The findings of this study indicate that CMs scaffolds are degradable and both in vitro and in vivo results indicate that the coating can prevent and delay the degradation of underlying Bio-CaP, even though the coating is biodegradable.

The coating and Bio-CaP were all biomimetically formed by precipitation of calcium phosphate. However, their surface structures were totally different before coating. It was demonstrated that BMP-2 incorporated into biomimetic coatings can retain its biological activity and that the growth factors released through osteoclastic activity could significantly promote osteoblastogenesis in vitro. Therefore, we assume that BMP-2 maintains its activity. However, further investigations are needed to prove the specific activity of BMP-2.

In bone tissue engineering, osteoblast cells are one of the most crucial factors that lead to the ultimate formation of new bone tissue. The ECM proteins function as a substratum for bone cell adhesion and serve as a scaffold for mineralization [18]. In the current work, the constructs of the CMs scaffold drug delivery system of DBB/CMs/BMP-2 were observed with SEM after culturing with an osteogenesis-inducing medium for 7 days (Figure 6). In general, cells growing on the DBB/CMs scaffold revealed many typical globular round cells which integrated with the scaffold. In particular, the DBB/CMs/BMP-2 group showed that the cells growing on this material were surrounded by a thick substance. The reason is that the BMP-2 produced many substances on the surface of the original scaffold, which assisted the osteoblasts growth. Besides, BMP-2 also assisted the proliferation tendency of the osteoblasts induced by the drug delivery scaffold (Figures 8 and 9).

Our findings strongly suggest that this CMs scaffold can be used as an effective protein delivery vehicle. In addition, the use of the coating can offer an alternative for slow release. CMs scaffolds can offer a dual-release system for a sequential delivery of different proteins/drugs. This combination could be applicable for a variety of clinical applications. For example, osteogenic agents can be incorporated into the interior of CMs scaffolds, and at the same time, antibiotics can be incorporated into the surface coating. This could be considered as a new strategy for the treatment of bone defects caused by peri-implantitis. In conclusion, it was shown that the CMs scaffold with an internal or surface-coated depot of protein has the capacity to maintain a slow and sustained protein release in the presence of osteoclasts in vitro. Further investigations are needed to prove the in vivo release and actual activity of BMP-2. The dual-drug release system offers a promising tool for the controlled delivery of multiple therapeutic agents, such as antibiotics, osteogenic agents, and anti-cancer drugs for different clinical applications.

## Conclusions

A functional scaffold loading microspheres system was successfully developed and shown to be an excellent drug delivery platform for bone regeneration. BMP-2 as a model bioactive molecule was efficiently added to the microsphere-loading DBB scaffold with perfect biocompatibility, and offered efficient release kinetics of BMP-2. Therefore, this novel CS microsphere-loaded DBB scaffold system may be applicable as a promising scaffold for bone regeneration.
